# Supplementary material for: Exercise and Interorgan Communication: Short-Term Exercise Training Blunts Differences in Consecutive Daily Urine 1H-NMR Metabolomic Signatures between Physically Active and Inactive Individuals
Source: Metabolites. 2022 May 24;12(6):473. doi: 10.3390/metabo12060473 (PMC9229485; doi:10.3390/metabo12060473)
Supplement: Supplementary file 1 [file metabolites-12-00473-s001.zip › File S1.pdf]

Electronic Supplementary Material S1

X-Adapt project

Manuscript title: Exercise and inter-organ communication: Short-term exercise training blunts differences in consecutive daily urine 1H-NMR metabolomic signatures between physically active and inactive individuals

Authors: Leon Deutsch 1, Alexandros Sotiridis 2,3, Boštjan Murovec4, Janez Plavec5, Igor Mekjavic3, Tadej Debevec,3,6 and Blaž Stres1,3,7*

[1] University of Ljubljana, Biotechnical Faculty, Department of Animal Science, Ljubljana, Slovenia;
[2] National and Kapodistrian University of Athens, School of Physical Education and Sport Science, Section of Sport Medicine and Biology of Exercise, Athens, Greece;
[3] Jožef Stefan Institute, Department of Automation, Biocybernetics and Robotics, Ljubljana, Slovenia;
[4] University of Ljubljana, Faculty of Electrical Engineering, Jamova 2, SI-1000 Ljubljana, Slovenia;
[5] National Institute of Chemistry, NMR Center, Ljubljana, Slovenia;
[6] University of Ljubljana, Faculty of Sports, Ljubljana, Slovenia;
[7] University of Ljubljana, Faculty of Civil and Geodetic Engineering, Institute of Sanitary Engineering, Ljubljana, Slovenia
* Correspondence: blaz.stres@bf.uni-lj.si; Tel.: +38641567633

A)

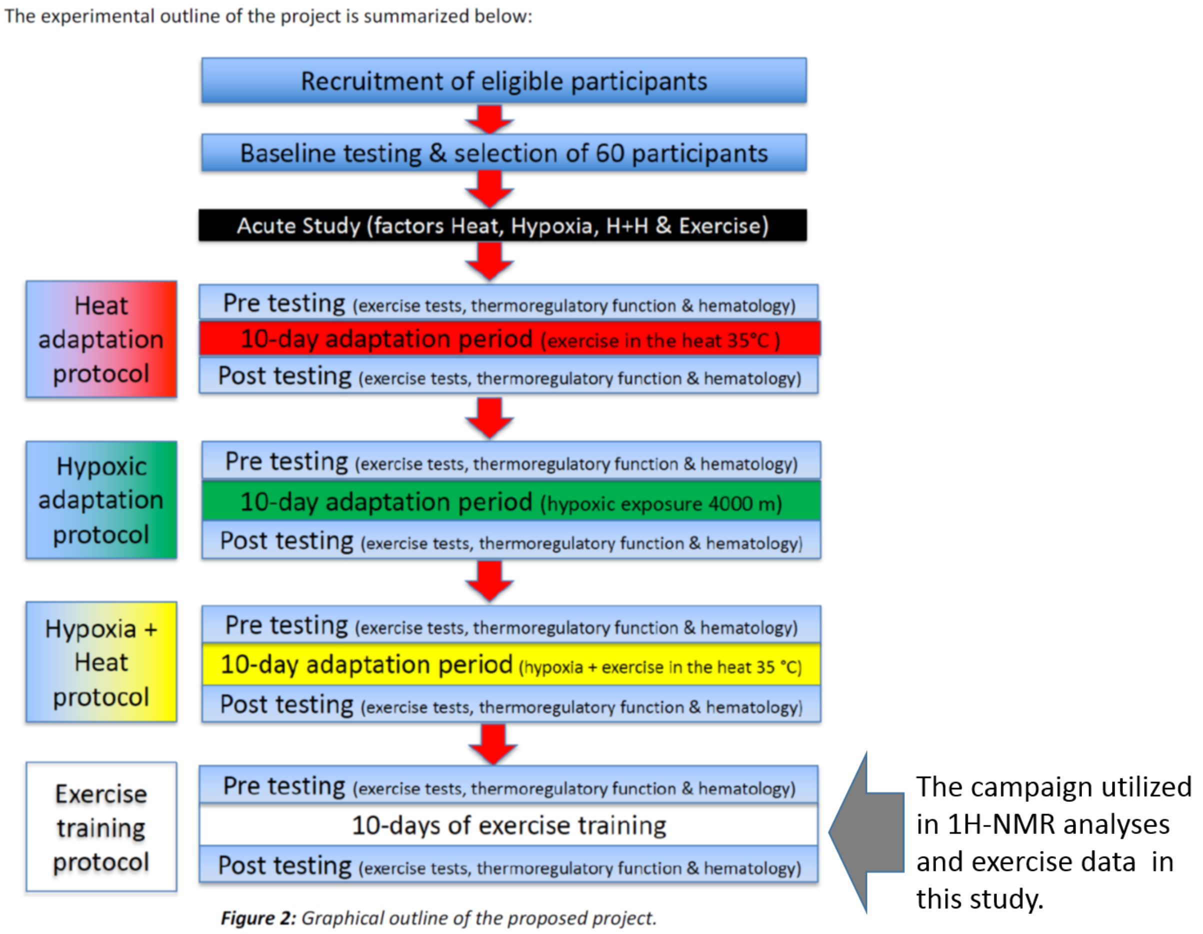

B)

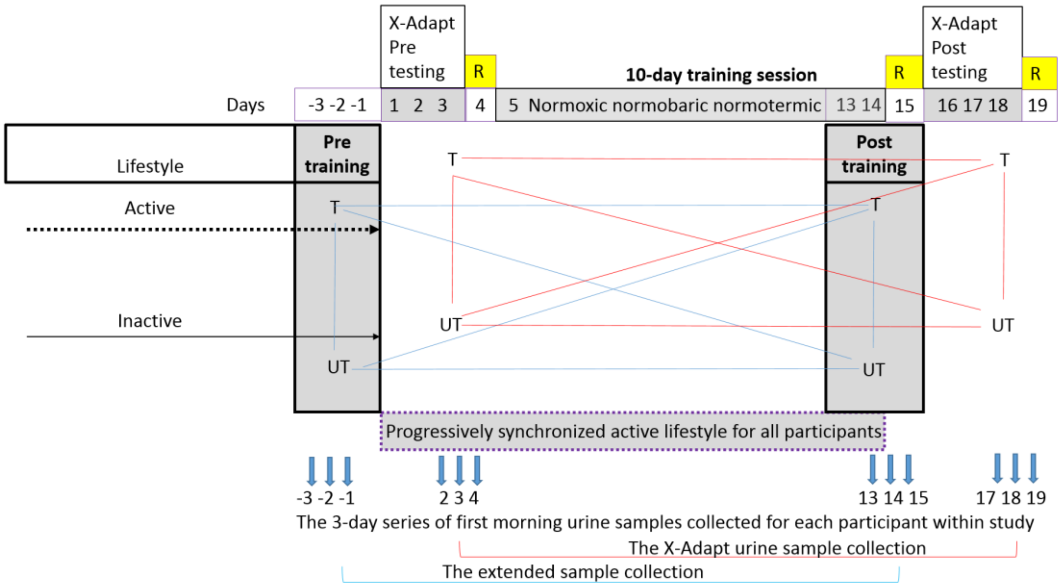

Figure S1. Schematic overview of the X-Adapt project campaigns as described before [1-4]. Arrow designates the campaign utilized in analyses in this study **(A)**. **(B)** A detailed representation of sampling protocol and distribution of subsections within the campaign. The vertical arrows designate the 3-day sampling sessions of morning urine. Horizontal lines designate the comparisons between groups of metabolic fingerprints analyzed in this study. Red lines: X-Adapt collection; Blue lines: Extended sample collection.

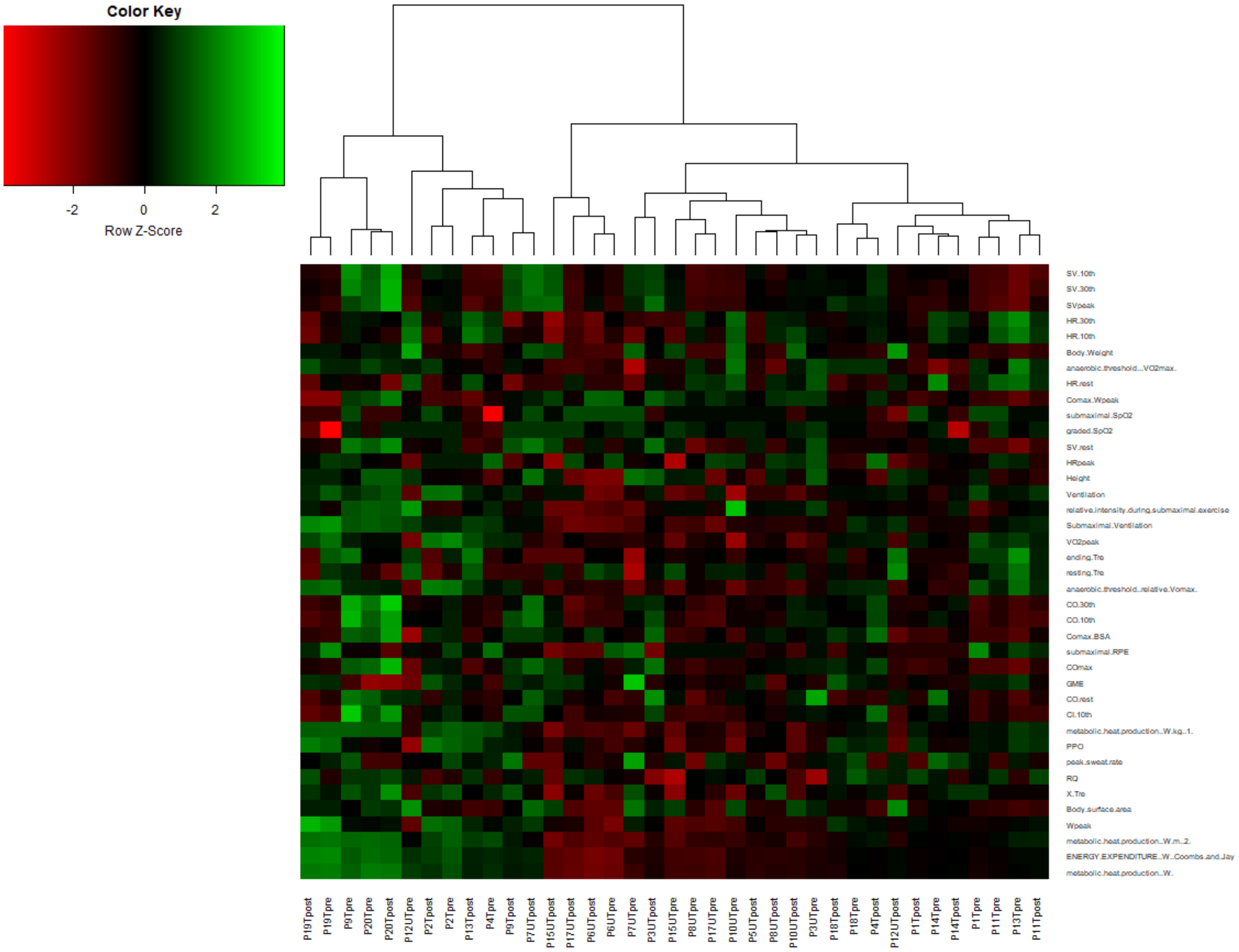

Figure S2. Physical characteristics of trained (T) and untrained (UT) participants involved in X-Adapt project pre testing (pre) and post testing (post). Abbreviations: SV=stroke volume; HR=hearth rate; COmax=cardiac output; CI=cardiy index; Tre=rectal temperature; Wpeak=maximal power output; PPO=peak power output; GME=gross mechanical efficiency

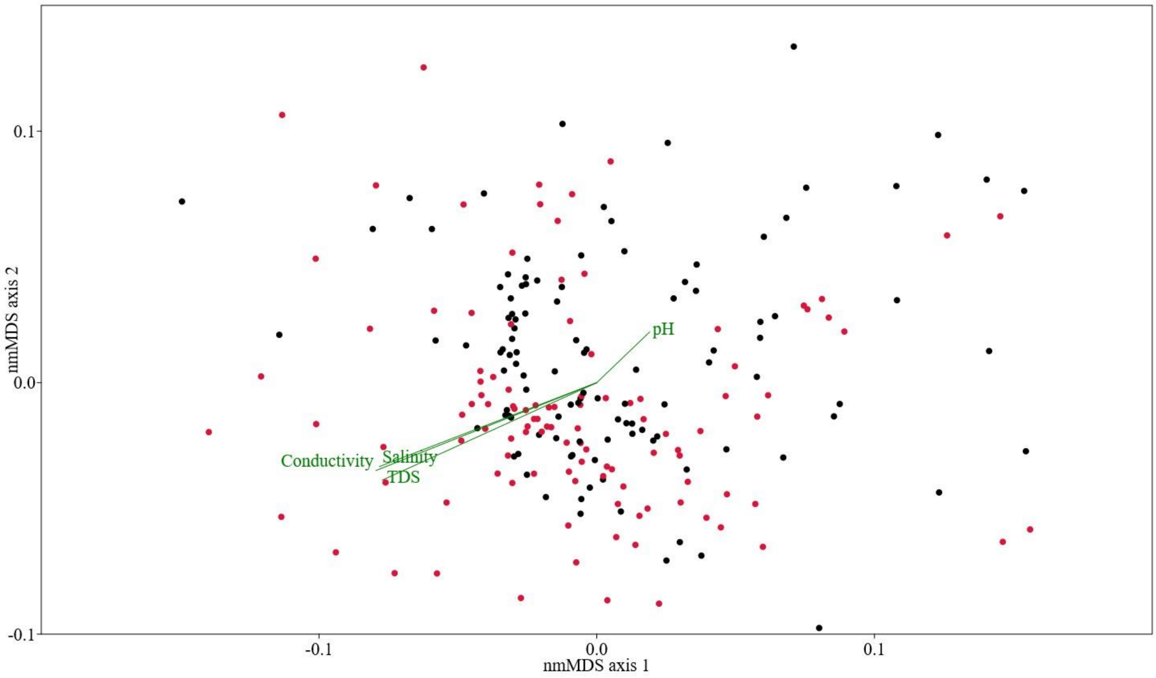

Figure S3. X-Adapt nmMDS ordination representing data scatter and the explanatory parameters (pH, salinity, total dissolved solids (TDS), participants) measured in urine samples. Dots: **black**-trained participants, **red**-untrained participants. nmMDS stress value: 0.4561.

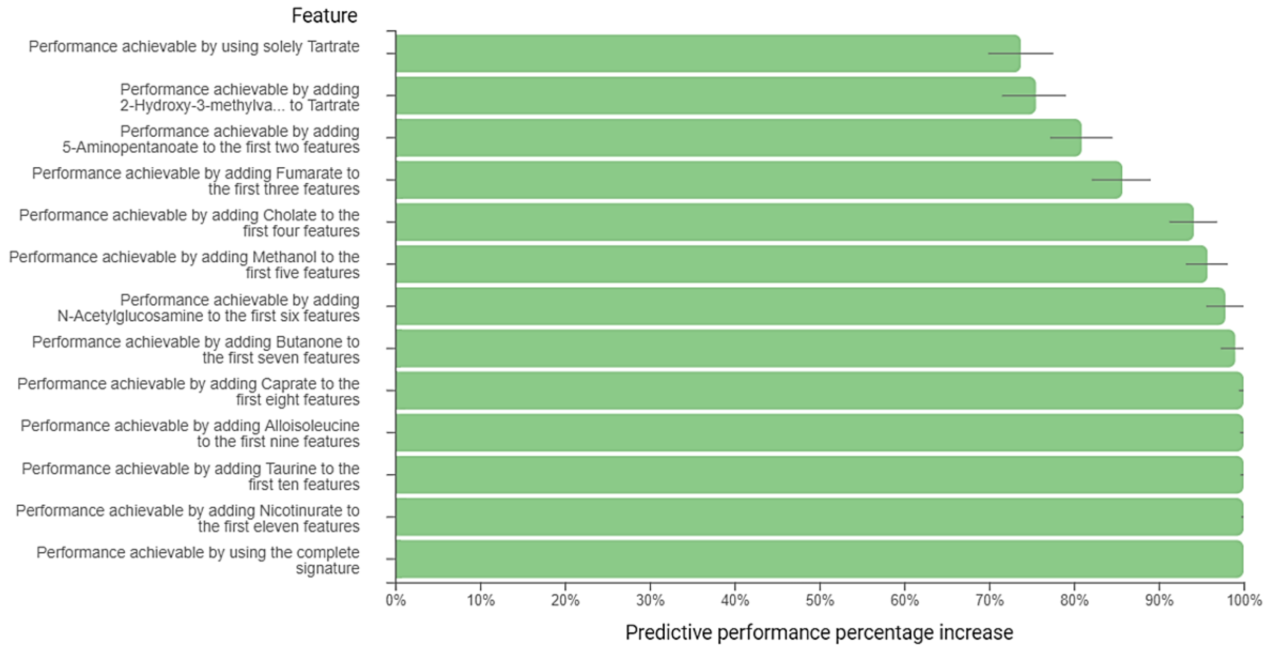

Figure S4. Predictive performance of the highest scoring models generated in this study, depending on the number of included relevant variables.

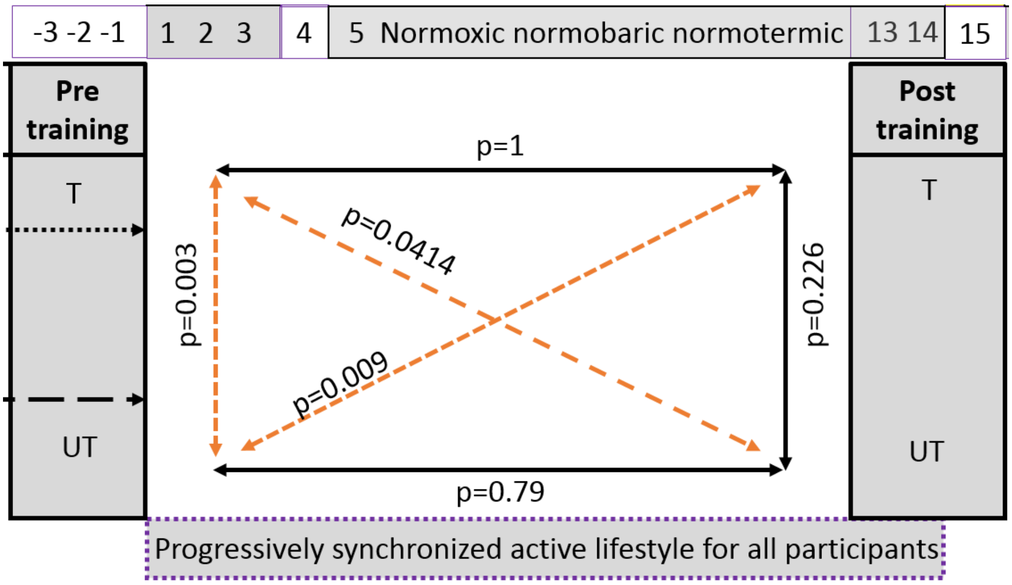

Figure S5. Schematic representation of npPERMANOVA (npermutations=5000) analysis. 10 day od activity at Wpeak of 50% led to unsignifficant differences between untrained and trained participant of the X-Adapt study.

Table S1. Sum of concentrations and numbers of metabolites observed in all groups.

**Untrained**	**pre**		**post**	
	Concentrations	Number	Concentrations	Number
**Average**	27219.8	173.3	25596.3	170.3
**SD**	7311.2	15.1	7051.9	16.8
**n**	52.0	52.0	58.0	58.0
**SEM**	1013.9	2.1	926.0	2.2
**Trained**	**pre**		**post**	
	Concentrations	Number	Concentrations	Number
**Average**	25681.2	168.1	27341.2	168.3
**SD**	6767.0	15.7	8045.3	15.2
**n**	50.0	50.0	60.0	60.0
**SEM**	957.0	2.2	1038.6	2.0

Pre= metabolites measured before tests, Post= metabolites measured after tests, SD=standard deviation, n=number of samples, SEM= standard error of the mean

Table S2. The most important features for discriminating trained from untrained group obtained by JADBIO machine learning approach.

**Feature**
Tartrate
2-Hydroxy-3-methylvalerate
5-Aminopentanoate
Fumarate
Cholate
Methanol
N-Acetylglucosamine
Butanone
Caprate
Alloisoleucine
Taurine
Nicotinurate
pH

Table S3: Group characteristics of individuals tested in this study measured and published before [1-4] and their matched urinary physico-chemical parameters (bold) (measured in this study) (* significant different comparing to trained group; ** significantly different according to Mann-Whitney test for equal means).

Group	Trained (n=10)	Untrained (n=10)
**Age (years)**	23 (2)	25 (3)
**Height (cm)**	180 (5)	179 (3)
**Mass (kg)**	74 (3)	85 (14)*
**BSA (m^2)**	1.96 (0.08)	2.05 (0.17)
**Body fat (%)**	9.2 (2.3)	16.3 (4.9)*
**VO_{2peak} ($ml \cdot kg^{-1} \cdot min^{-1}$)**	58 (6)	42 (5)*
**W_{peak} (W)**	364 (35)	309 (46)*
**W_{peak}/kg (W/kg)**	4.9 (0.5)	3.6 (0.4)*
**Salinity (%)**	**0.55 (0.2)**	**0.57 (0.2)****
**pH**	**3.7 (0.7)**	**3.5 (0.9)****
**Conductivity (mS)**	**11.2 (3.8)**	**11.6 (4.0)****
**Total dissolved solids (ppt)**	**8.4 (3.4)**	**8.8 (3.5)****

BSA=body surface area, VO_{2peak}=maximal oxygen uptake, W_{peak}=maximal power output, W_{peak}/kg=maximal power output per kg of body mass

Instructions for running a model on a local machine

JADBIO allows the user to download a model and run it on a local machine.

To run our model locally, the user must meet the following requirements:

1. Java SE Development Kit version 17 (https://www.oracle.com/java/technologies/javase/jdk17-archive-downloads.html)

2. the Java executor (contained in model.zip - filename: jadbio-1.4.55-model-exe.jar)

3. model (contained in model.zip - filename: jadbio-1.3.31-model.bin)

After installing Java SE JDK, model.zip must be saved somewhere on the local machine. After saving model.zip, provided by the authors, the folder must be extracted (e.g. with WinZip, 7zip). The model must be executed with the command prompt (cmd) (Fig. 1, 2).

Step 1

Using the `cd path` command (Fig. 1), the user navigates to the same directory (e.g. Folder) that contains the model executor (.jar) and the model (.bin).

```
Select Ukazni poziv
Microsoft Windows [Version 10.0.19043.1586]
(c) Microsoft Corporation. Vse pravice pridržane.

C:\Users\LDeutsc>cd C:\Users\LDeutsc\Desktop\model
```

Figure 1. First command to navigate to the folder containing the model. In this case, we used the `cd C:\Users\LDeutsc\Desktop\model` command because Executor and Model were in the model folder on the desktop.

jadbio-1.3.31-model.bin	14. 05. 2022 20:47	Datoteka BIN	23 KB
jadbio-1.4.55-model-exe.jar	14. 05. 2022 20:47	Executable Jar File	11.249 KB

Figure 2. The two files needed for the overview of the model. Both files are contained in the model.zip folder.

Step 2

The next step is to preview the model using the following command:

```
Java --enable-preview -jar jadbio-1.4.55-model-exe.jar -n jadbio-1.3.31-model.bin
```

This allows the user to get an overview of the model, key features and information about the analysis (which algorithm was used, version of JADBIO and other information about the model) (Fig. 3).

```
Model:
  Ridge Logistic Regression Model

Signature variables:
  Cholate,Tartrate,5-Aminopentanoate,Caprate,Methanol,2-Hydroxy-3-methylvalerate,Butanone,Fumarate,Alloisoleucine,N-Acet
ylglucosamine,Nicotinurate,pH,Taurine

Analysis info:
  id=16079, title='trained untrained jadbio FK.training_Activity', type='CLASSIFICATION', target='Activity', dataset='tr
ained untrained jadbio FK.training', dataset_id='17133', project='XADAPT', metric='AUC', feature_selection=true, interpr
etable=false, max_vars=25, max_signatures=null
```

Figure 3. Overview of the model.

Step 3

To test the model, the user must prepare data. For the model to work properly, the concentrations must be in µM. The user needs to prepare the dataset as shown in Figure 4 and save it in a .csv document (comma separated values). After preparation, the prepared dataset must be saved in the same directory as the model executor and the model itself.

A	B	C	D	E	F	G	H	I	J	K	L	M	N
Sample	pH	Tartrate	2-Hydroxy	5-Aminope	Fumarate	Cholate	Methanol	N-Acetylgl	Butanone	Caprate	Alloisoleuc	Taurine	Nicotinurate
xadapt_001	3.31	3	0	0	0	0	8	0	0	21.5	0	102.8	0
xadapt_002	3.83	0	0	0	0	1	1.9	0	0	0	0	0	0
xadapt_003	5.52	0.8	0	0	0	0	4.1	0	0	0	0	0	0
xadapt_004	3.6	0	0	0	1.2	0.3	6.9	0	0	0	0	0	0
xadapt_005	4.87	10.1	0	0	0	0.3	15.4	44.8	0	0	0	42.6	0
xadapt_006	3.78	8.8	0	0	0	0.3	3.4	35.1	0	0	0	0	0
xadapt_007	4.45	4.8	0	0	0	0	3.1	0	0	0	1.4	0	0
xadapt_008	3.88	0	0	0	0	0	12.6	0	0	0	0	0	0
xadapt_009	3.62	1.9	0	0	0	0	0	0	0	0	0	0	0
xadapt_011	3.74	2.8	0	0	0	0	7.2	0	4.8	0	0	0	0
xadapt_012	3.98	20.6	0	0	0	0.4	0	0	0	0	0	0	0
xadapt_013	3.82	0	0	0	0	0	0.8	0	0	11.3	0	0	0
xadapt_014	3.23	4.7	0	0	0	1.6	5.1	15	0	0	0	0	0

Figure 4. Prepared dataset.

Step 4

After saving the dataset, the user must use the next command in the terminal:

```
java --enable-preview -jar jadbio-1.4.55-model-exe.jar -m jadbio-1.3.31-model.bin -i test1.csv -o result.csv
```

This command runs the model on test data (MPtest.csv in our case) and creates a new dataset with predictions (MPtest-output.csv) (Fig. 5).

```
C:\Users\LDeutsc\Desktop\model>Java --enable-preview -jar jadbio-1.4.55-model-exe.jar -m jadbio-1.3.31-model.bin -i test1.csv -o result.csv
Successfully loaded model from jadbio-1.3.31-model.bin
Successfully loaded input dataset from test1.csv
Successfully wrote predictions to result.csv
```

Figure 5. Executing the model and creating the output .csv file with predictions in the same directory.

Step 5

After model execution, the user can check the calculated predictions by opening the .csv file directly by clicking on the created .csv file and opening it in any data analysis program (Excel, Past, R ...). As shown in Fig. 6 the model classifies the data between different classes (MP1, MP2 and MP3). The first column is the same as in the test data created by the user. For issues with classification of MP2 class please see also Figure 2 in the original publication (Deutsch and Stres, 2021)

Sample name	Prob (class = trained)	Prob (class = untrained)
xadapt_001	0.694572528	0.305427472
xadapt_002	0.815910454	0.184089546
xadapt_003	0.955969117	0.044030883
xadapt_004	0.857383888	0.142616112
xadapt_023	0.798564526	0.201435474
xadapt_024	7.00E-06	0.999992999
xadapt_025	0.110582248	0.889417752
xadapt_026	0.360483234	0.639516766
xadapt_027	5.47E-31	1
xadapt_029	0.214583769	0.785416231
xadapt_030	7.17E-04	0.999282937
xadapt_031	0.582942833	0.417057167
xadapt_032	0.394083655	0.605916345
xadapt_033	2.21E-06	0.999997793
xadapt_034	3.87E-08	0.999999961

Figure 6. The newly created .csv file with predictions calculated from test data.

References

1. Sotiridis, A.; Debevec, T.; McDonnell, A.C.; Ciuha, U.; Eiken, O.; Mekjavic, I.B. Exercise cardiorespiratory and thermoregulatory responses in normoxic, hypoxic and hot environment following 10-day continuous hypoxic exposure. *Journal of applied physiology (Bethesda, Md. : 1985)* **2018**, *125*, 1284–1295, doi:10.1152/japplphysiol.01114.2017.
2. Sotiridis, A.; Debevec, T.; Ciuha, U.; Eiken, O.; Mekjavic, I.B. Heat acclimation does not affect maximal aerobic power in thermoneutral normoxic or hypoxic conditions. *Experimental physiology* **2019**, *104*, doi:10.1113/EP087268.
3. Sotiridis, A. Independent and Combined Effects of Heat and Hypoxic Acclimation on Exercise Performance in Humans: With Particular Reference to Cross-adaption: Doctoral Dissertation. Jozef Stefan Institute, Ljubljana, 2019.
4. Sotiridis, A.; Debevec, T.; Ciuha, U.; McDonnell, A.C.; Mlinar, T.; Royal, J.T.; Mekjavic, I.B. Aerobic but not thermoregulatory gains following a 10-day moderate-intensity training protocol are fitness level dependent: A cross-adaptation perspective. *Physiological reports* **2020**, *8*, doi:10.14814/phy2.14355.
