# Supplementary material for: Exercise and Interorgan Communication: Short-Term Exercise Training Blunts Differences in Consecutive Daily Urine 1H-NMR Metabolomic Signatures between Physically Active and Inactive Individuals
Source: Metabolites. 2022 May 24;12(6):473. doi: 10.3390/metabo12060473 (PMC9229485; doi:10.3390/metabo12060473)
Supplement: Supplementary file 1 [file metabolites-12-00473-s001.zip › File S3.pdf]

Example 1H-NMR spectra characteristic of the trained- and untrained groups

- Representative spectra from untrained and trained participant of the X-Adapt study before (pre) and after (post) performed exercise
- Water region was excluded

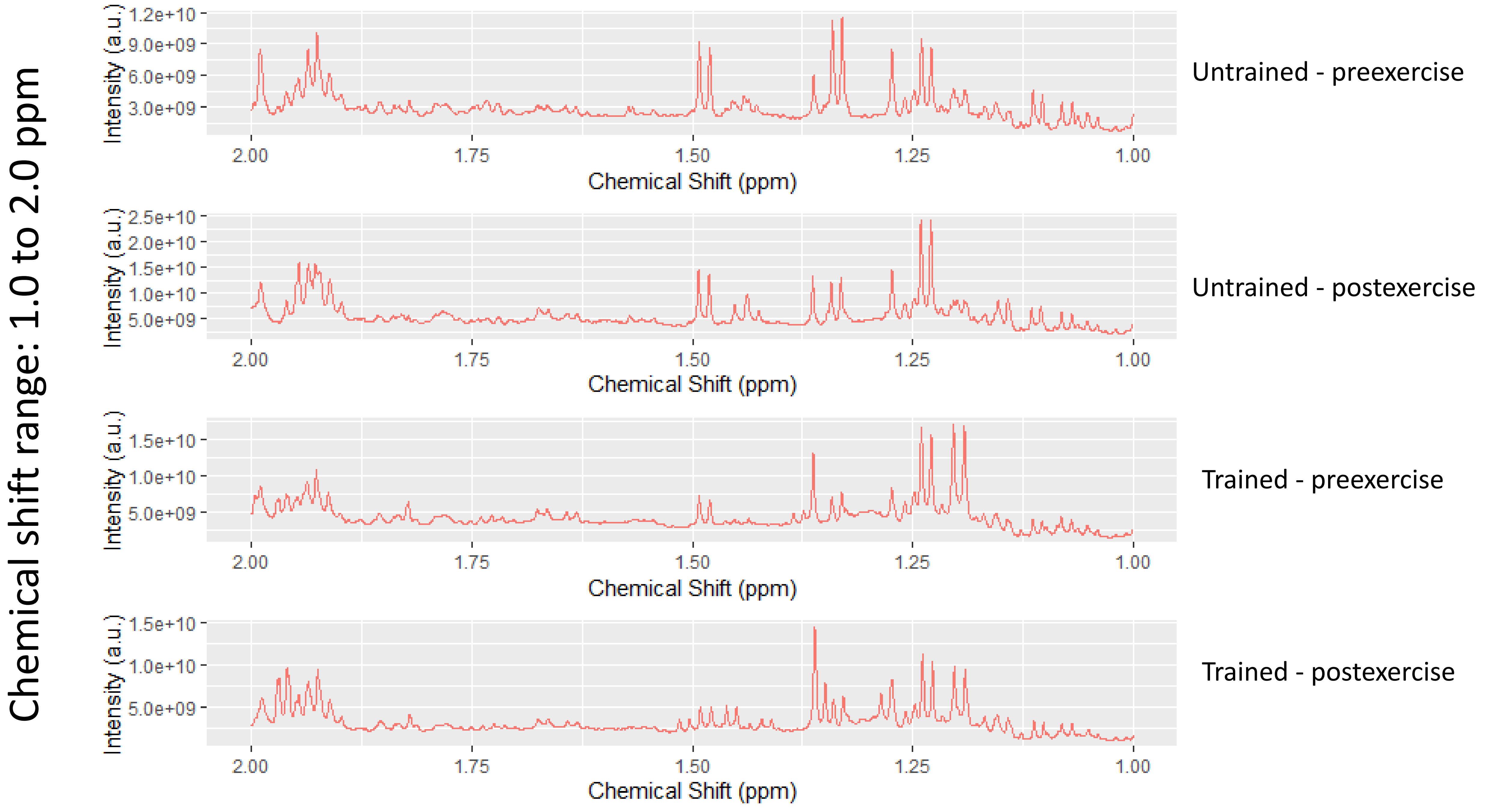

Chemical shift range: 1.0 to 2.0 ppm
Untrained - preexercise
Untrained - postexercise
Trained - preexercise
Trained - postexercise
Intensity (a.u.)
Chemical Shift (ppm)
1.2e+10
9.0e+09
6.0e+09
3.0e+09
2.5e+10
2.0e+10
1.5e+10
1.0e+10
5.0e+09
2.00
1.75
1.50
1.25
1.00

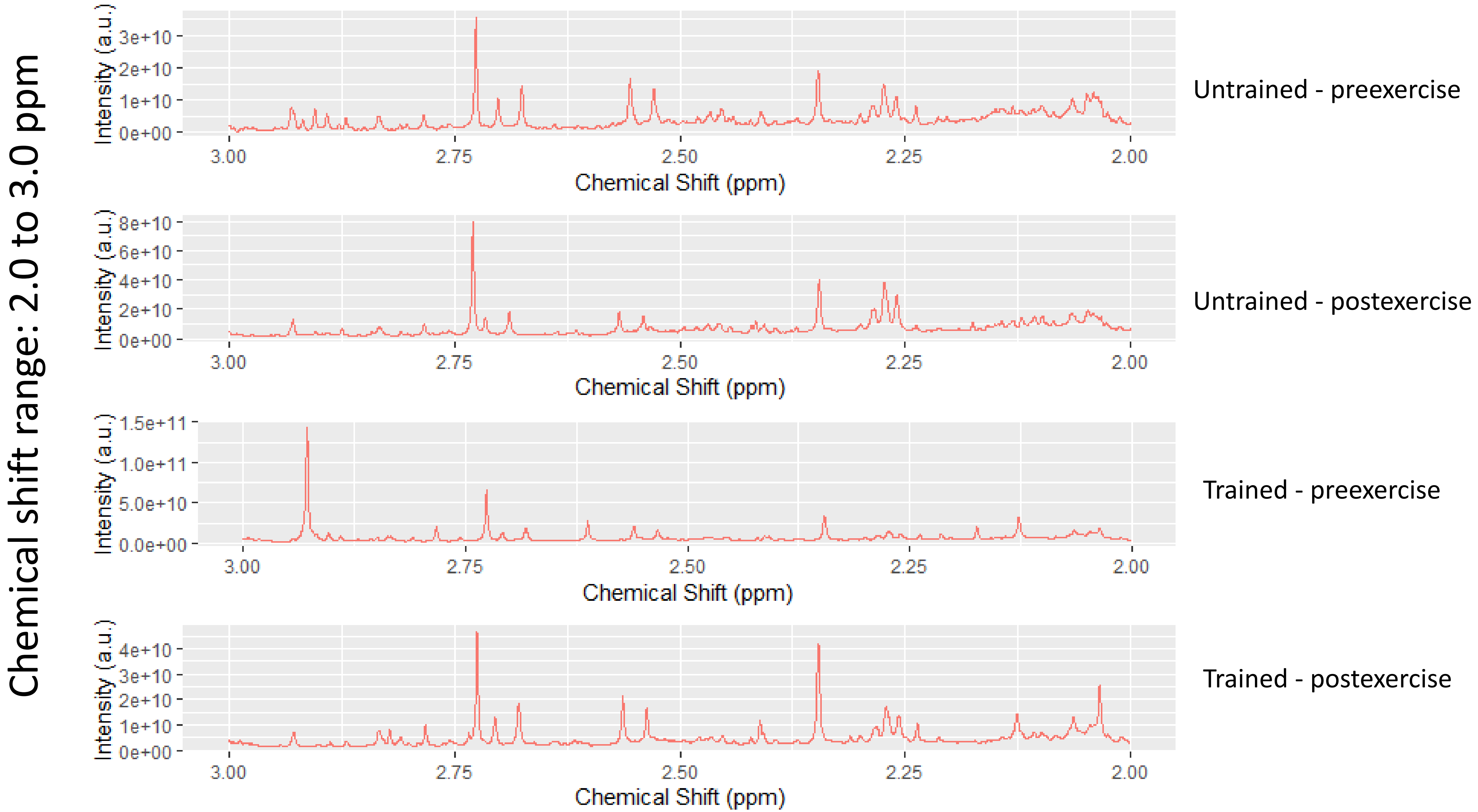
Chemical shift range: 2.0 to 3.0 ppm
Untrained - preexercise
Untrained - postexercise
Trained - preexercise
Trained - postexercise
Intensity (a.u.)
3e+10
2e+10
1e+10
0e+00
8e+10
6e+10
4e+10
2e+10
0e+00
1.5e+11
1.0e+11
5.0e+10
0.0e+00
4e+10
3e+10
2e+10
1e+10
0e+00
3.00
2.75
2.50
2.25
2.00
Chemical Shift (ppm)

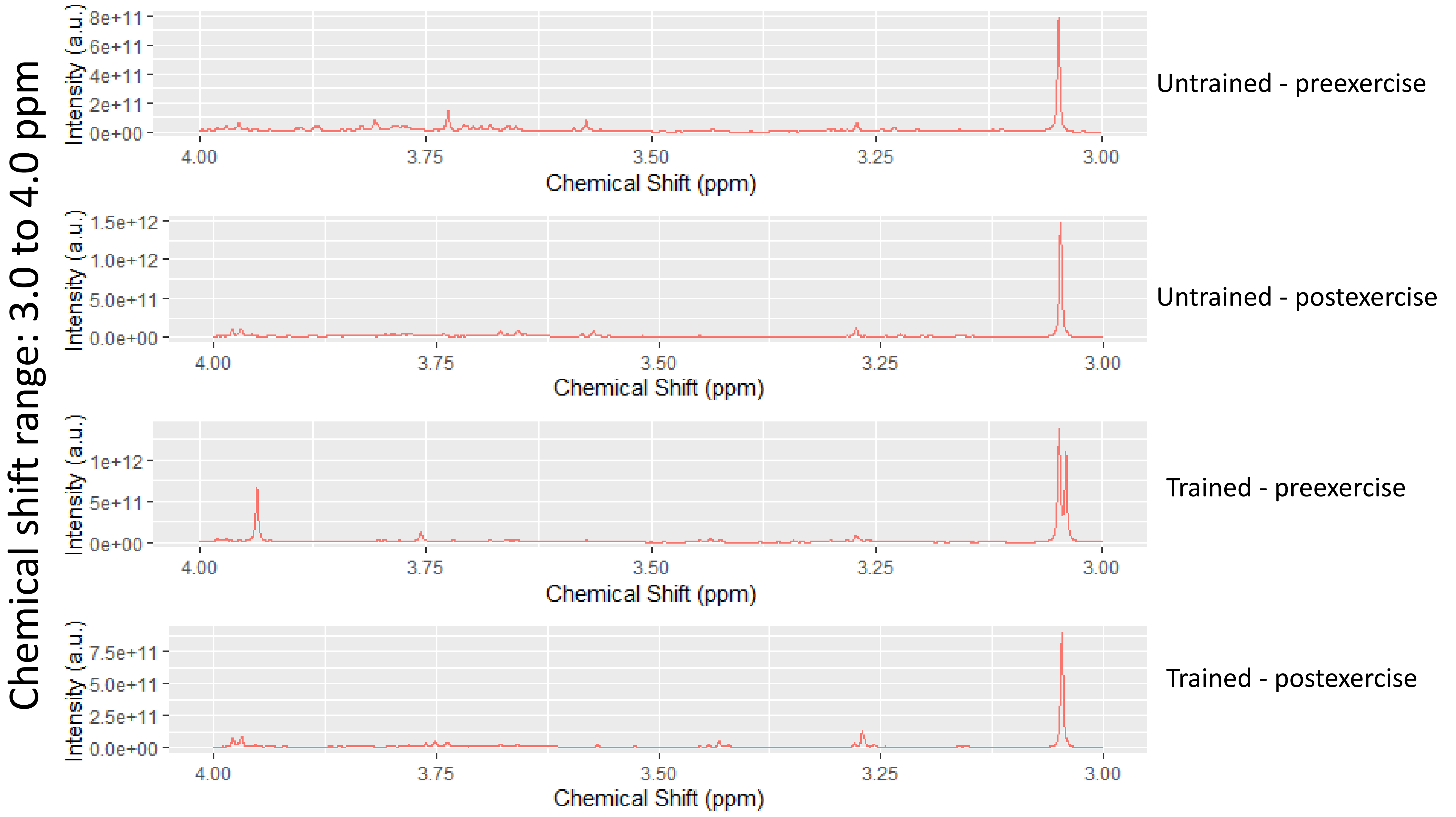
Chemical shift range: 3.0 to 4.0 ppm
Untrained - preexercise
Untrained - postexercise
Trained - preexercise
Trained - postexercise
Intensity (a.u.)
Chemical Shift (ppm)
4.00
3.75
3.50
3.25
3.00
0e+00
2e+11
4e+11
6e+11
8e+11
0.0e+00
5.0e+11
1.0e+12
1.5e+12
5e+11
1e+12
2.5e+11
7.5e+11

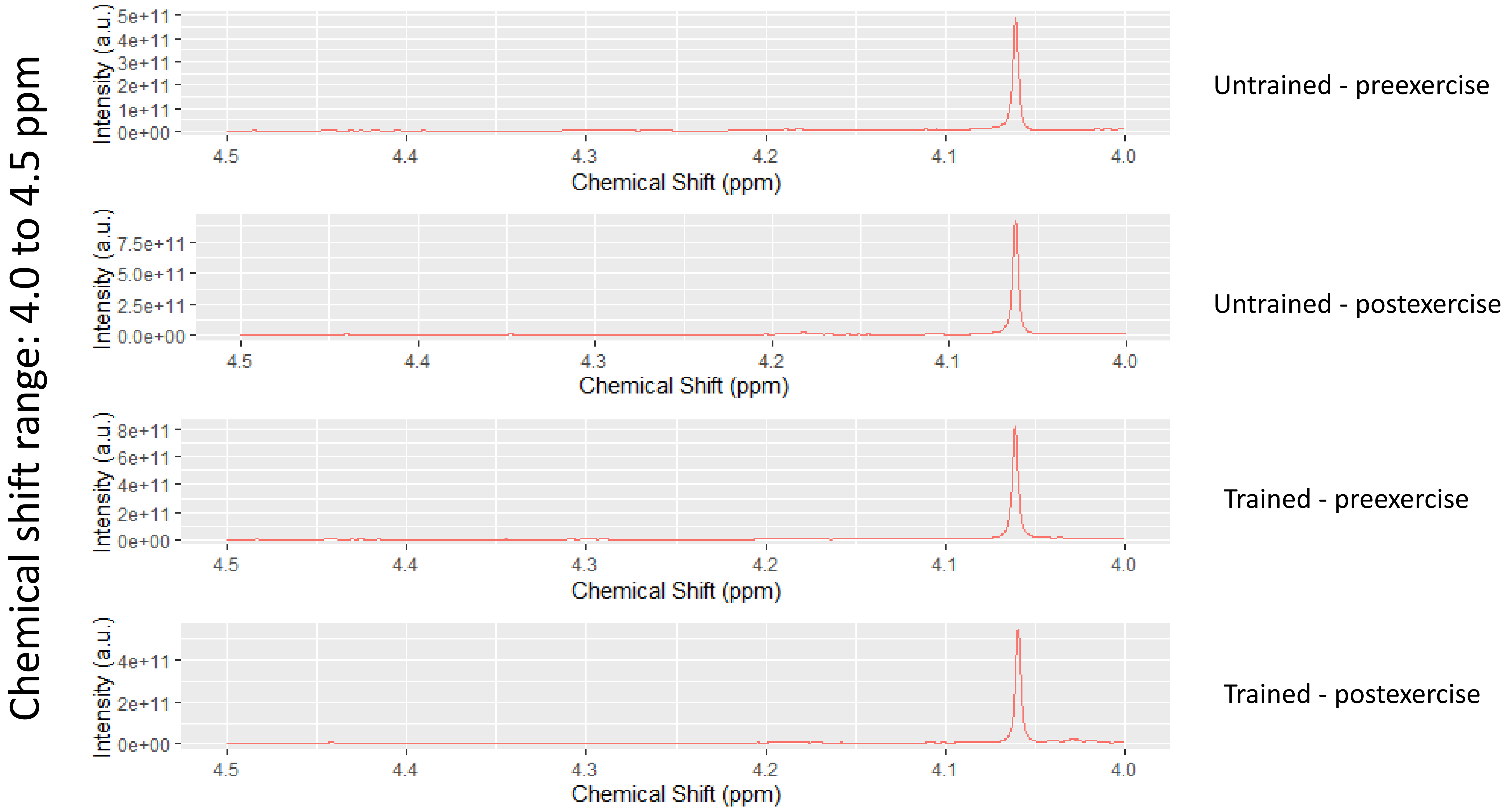
Chemical shift range: 4.0 to 4.5 ppm
Intensity (a.u.)
5e+11
4e+11
3e+11
2e+11
1e+11
0e+00
4.5
4.4
4.3
4.2
4.1
4.0
Chemical Shift (ppm)
Untrained - preexercise
7.5e+11
5.0e+11
2.5e+11
0.0e+00
Untrained - postexercise
8e+11
6e+11
4e+11
2e+11
0e+00
Trained - preexercise
4e+11
2e+11
0e+00
Trained - postexercise

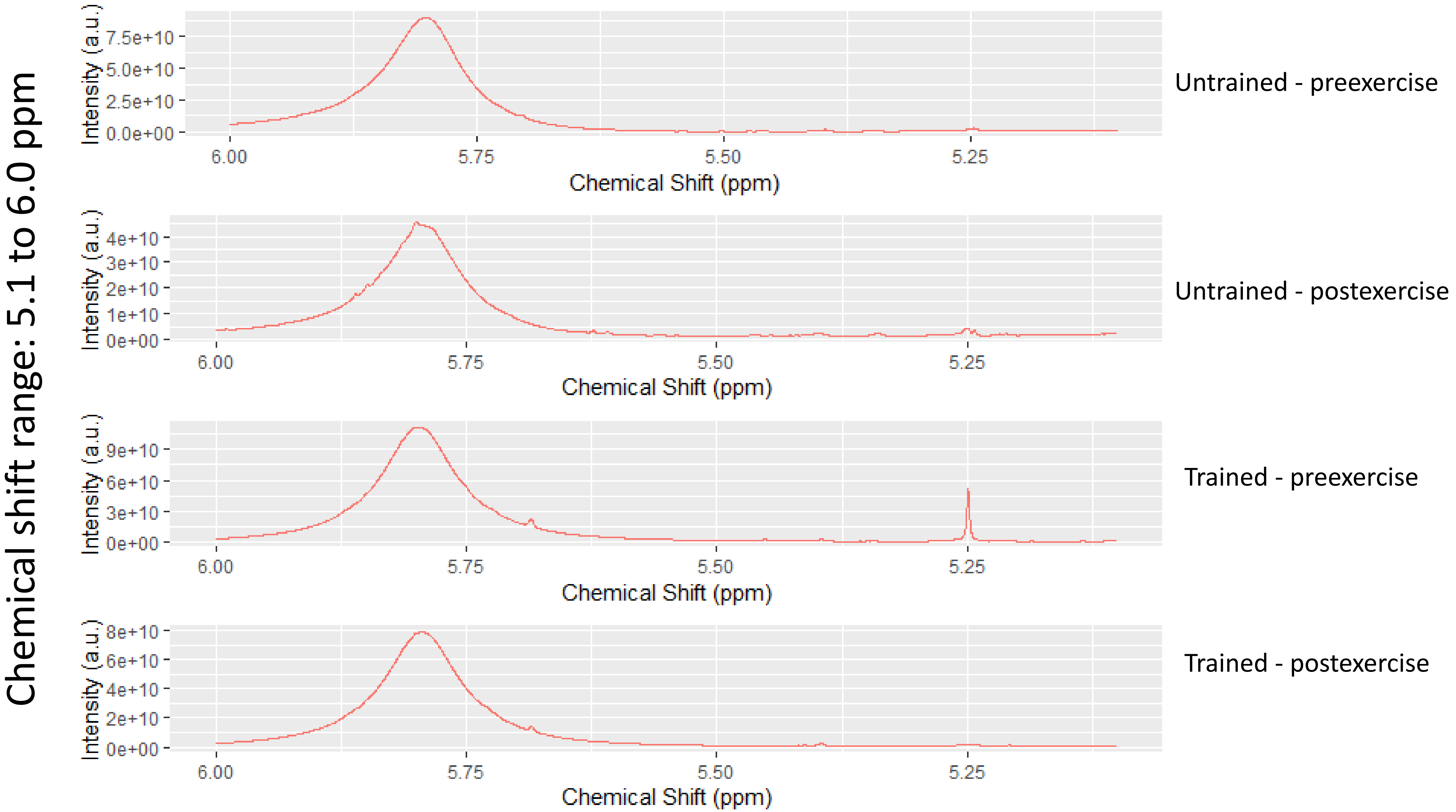

Chemical shift range: 5.1 to 6.0 ppm
Untrained - preexercise
Untrained - postexercise
Trained - preexercise
Trained - postexercise
Intensity (a.u.)
Chemical Shift (ppm)
6.00
5.75
5.50
5.25
7.5e+10
5.0e+10
2.5e+10
0.0e+00
4e+10
3e+10
2e+10
1e+10
0e+00
9e+10
6e+10
3e+10
8e+10
6e+10
4e+10

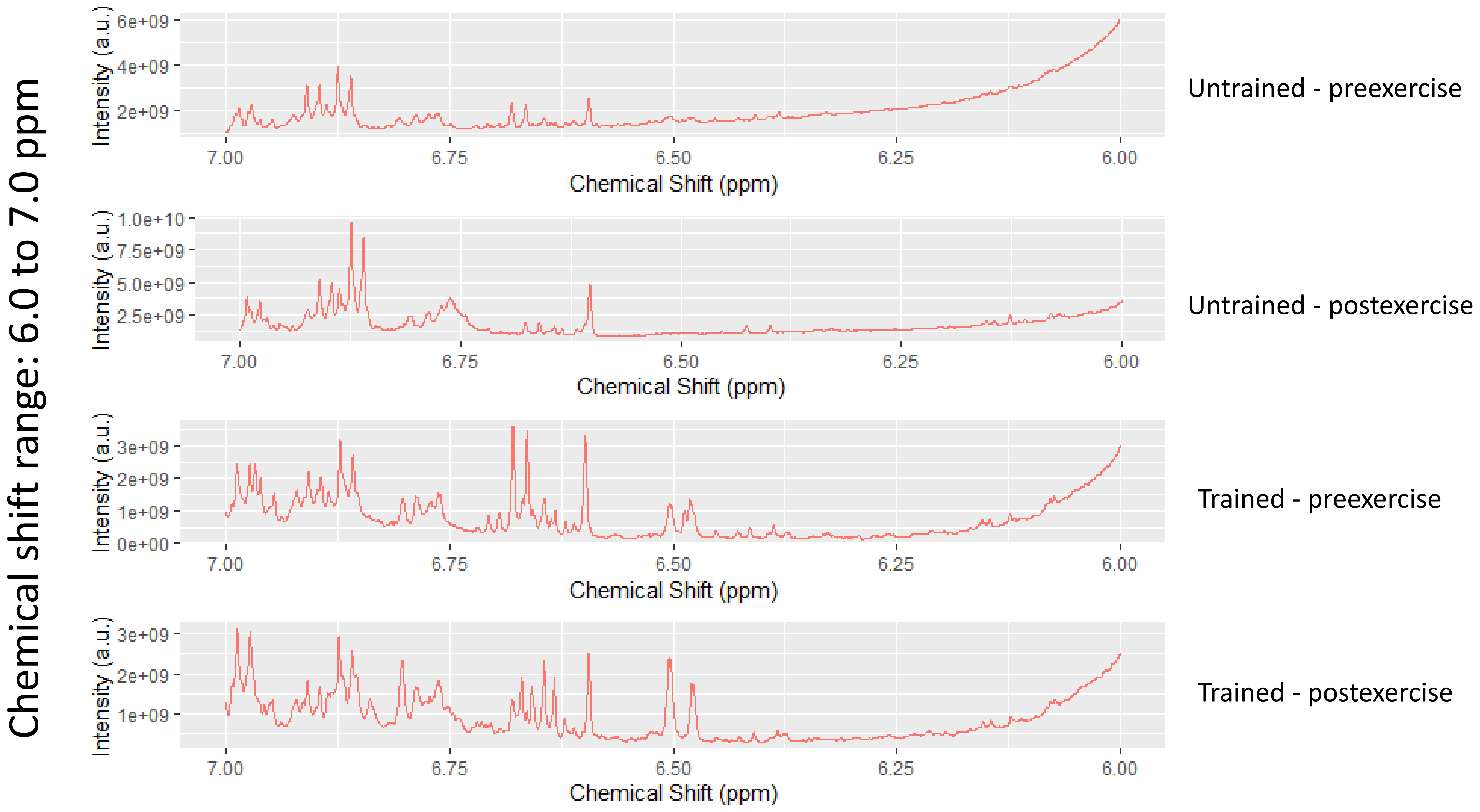
Chemical shift range: 6.0 to 7.0 ppm
Intensity (a.u.)
6e+09
4e+09
2e+09
7.00
6.75
6.50
6.25
6.00
Chemical Shift (ppm)
Untrained - preexercise
1.0e+10
7.5e+09
5.0e+09
2.5e+09
Untrained - postexercise
3e+09
2e+09
1e+09
0e+00
Trained - preexercise
Trained - postexercise

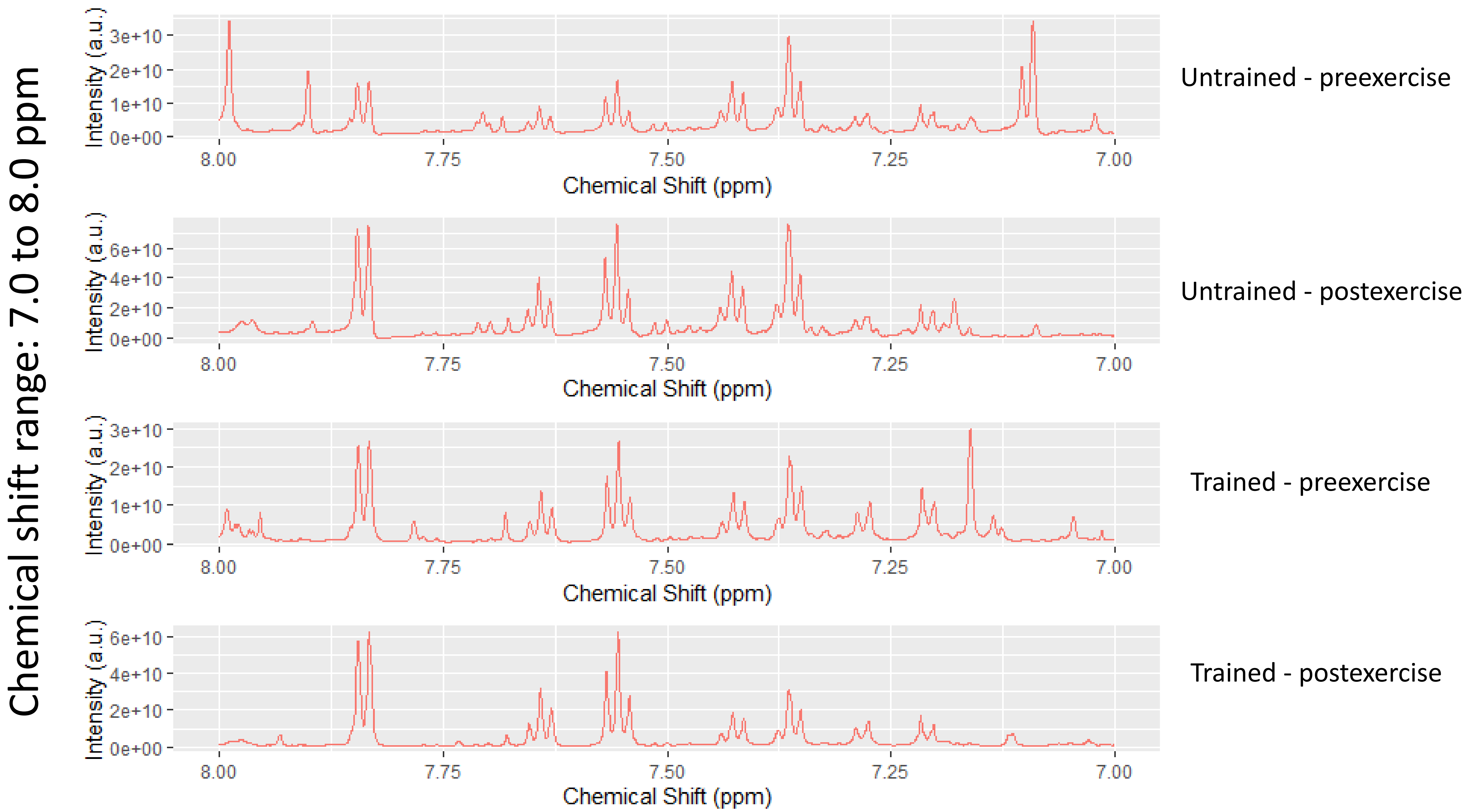

Chemical shift range: 7.0 to 8.0 ppm
Untrained - preexercise
Untrained - postexercise
Trained - preexercise
Trained - postexercise
Intensity (a.u.)
3e+10
2e+10
1e+10
0e+00
6e+10
4e+10
2e+10
0e+00
8.00
7.75
7.50
7.25
7.00
Chemical Shift (ppm)

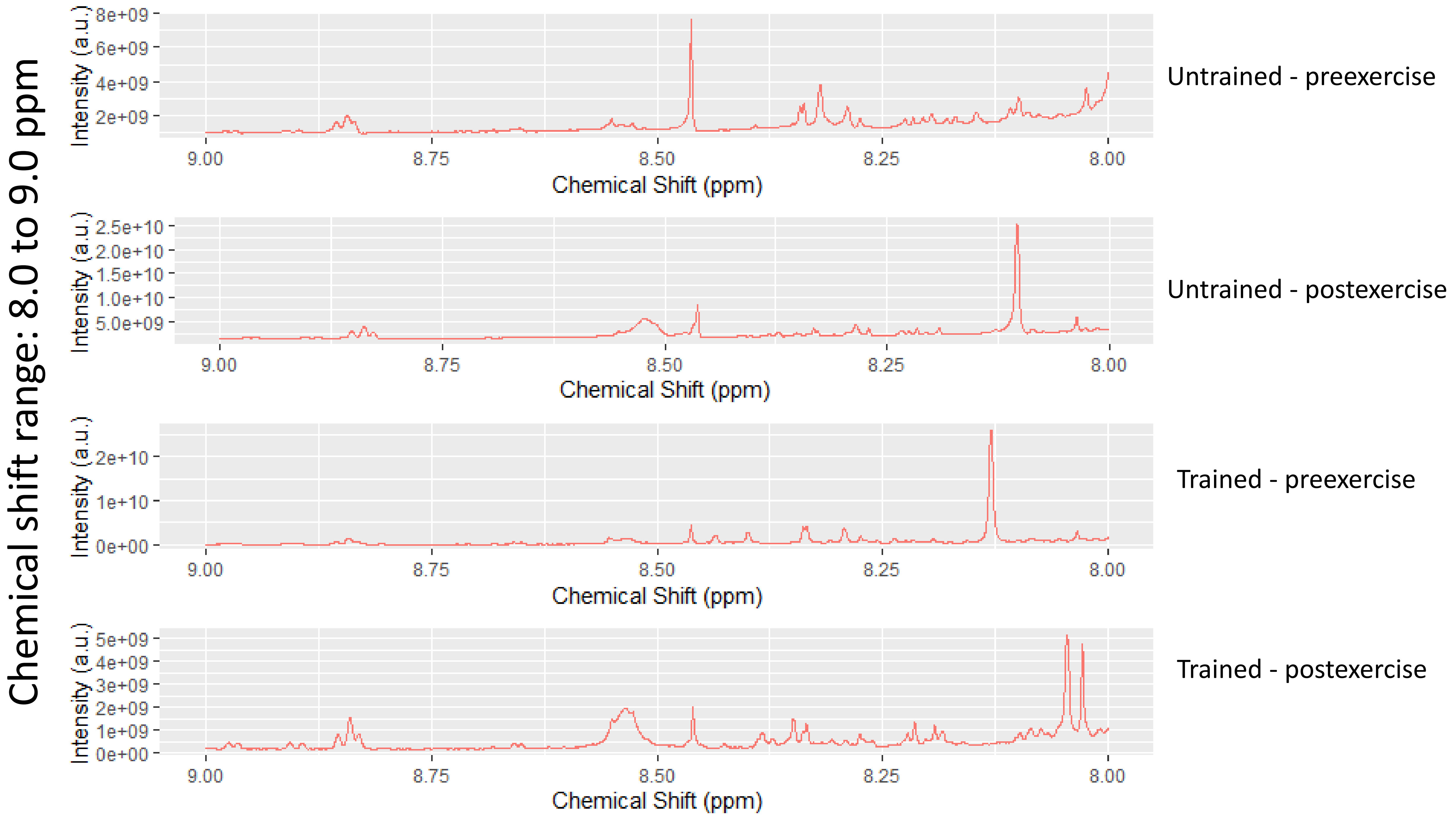
Chemical shift range: 8.0 to 9.0 ppm
Untrained - preexercise
Untrained - postexercise
Trained - preexercise
Trained - postexercise
Intensity (a.u.)
Chemical Shift (ppm)
9.00
8.75
8.50
8.25
8.00
8e+09
6e+09
4e+09
2e+09
2.5e+10
2.0e+10
1.5e+10
1.0e+10
5.0e+09
2e+10
1e+10
0e+00
5e+09
4e+09
3e+09
2e+09
1e+09

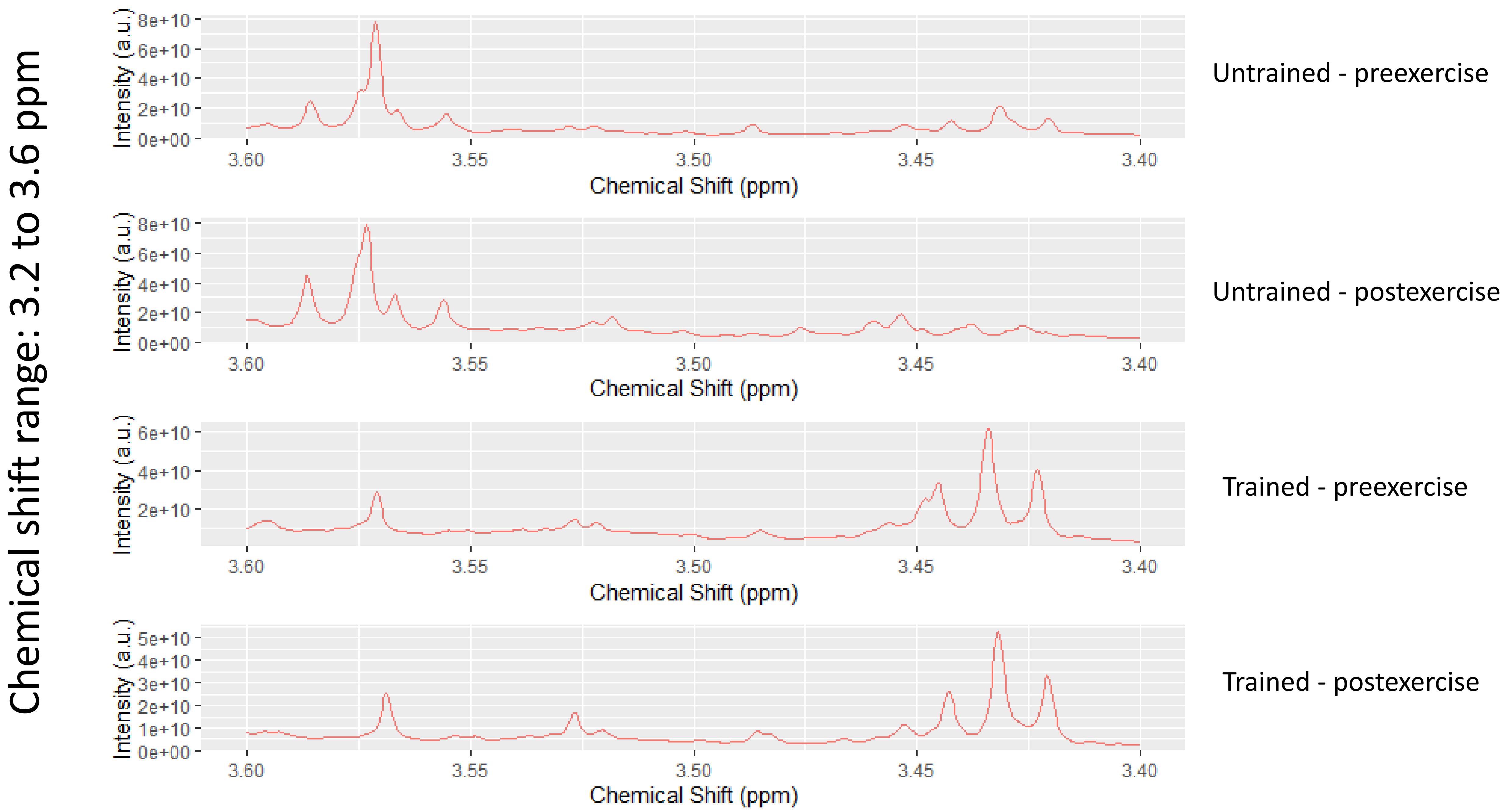
Chemical shift range: 3.2 to 3.6 ppm
Untrained - preexercise
Untrained - postexercise
Trained - preexercise
Trained - postexercise
Intensity (a.u.)
8e+10
6e+10
4e+10
2e+10
0e+00
5e+10
3e+10
1e+10
3.60
3.55
3.50
3.45
3.40
Chemical Shift (ppm)

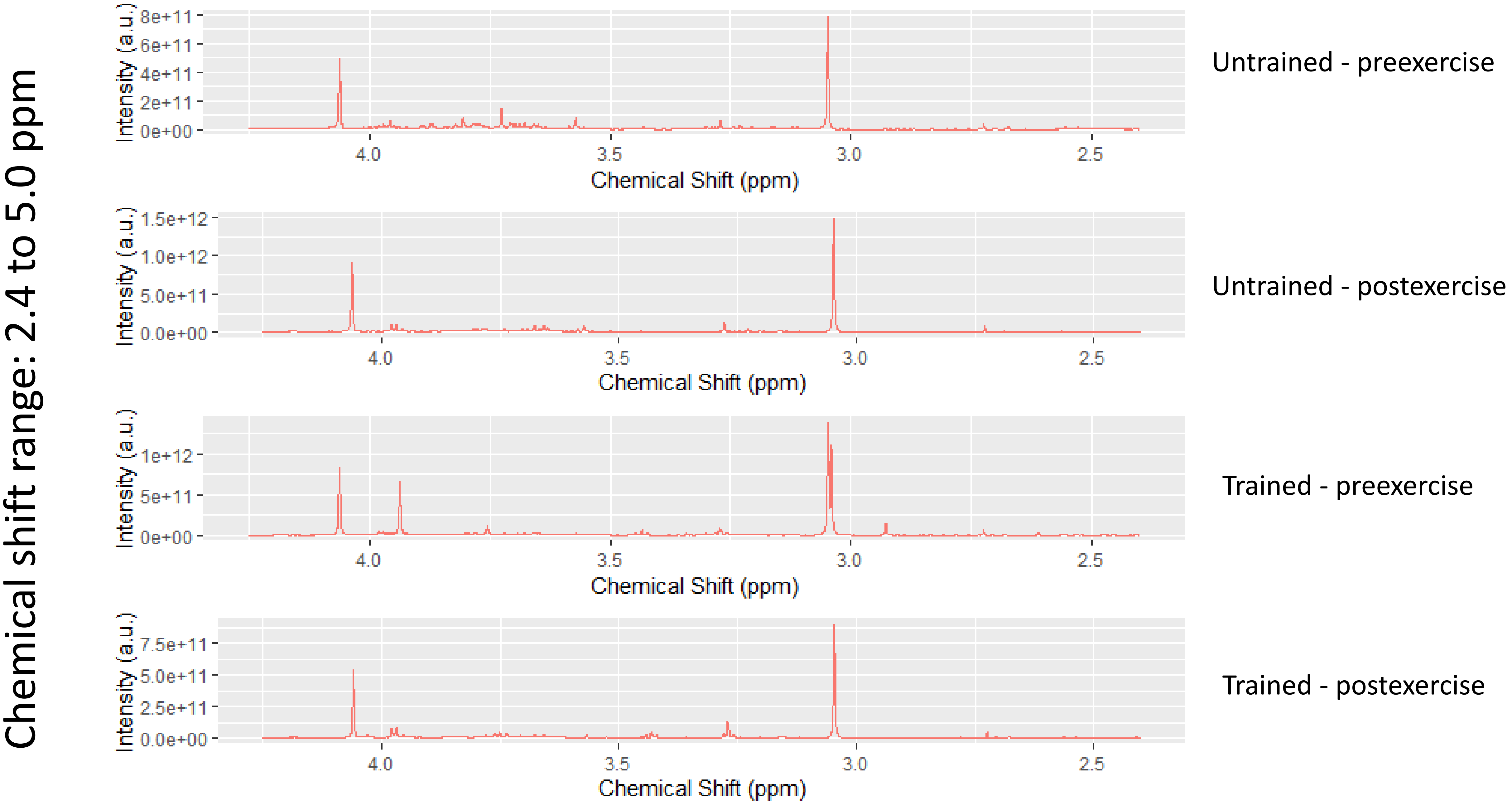
Chemical shift range: 2.4 to 5.0 ppm
Untrained - preexercise
Untrained - postexercise
Trained - preexercise
Trained - postexercise
Intensity (a.u.)
Chemical Shift (ppm)
8e+11
6e+11
4e+11
2e+11
0e+00
1.5e+12
1.0e+12
5.0e+11
0.0e+00
1e+12
5e+11
7.5e+11
2.5e+11
4.0
3.5
3.0
2.5
